# Medical Student Syndrome and Imposter Syndrome: Are They Real?

**DOI:** 10.31729/jnma.8531

**Published:** 2024-04-30

**Authors:** Deekshya Devkota

**Affiliations:** 1 Nepalese Army Institute of Health Sciences, Kathmandu, Nepal

**Keywords:** *hypochondriasis*, *imposter syndrome*, *medical student*

## Abstract

Medical Student Syndrome and imposter syndrome are rampant in the medical profession, especially among young medical students. Medical Student Syndrome, also known as the third-year syndrome is a hypochondriacal concern in inexperienced medical students regarding the disease they are studying. Usually, it arises as a result of incomplete knowledge about the disease and regular exposure to new information about diseases. Imposter syndrome, on the other hand, refers to the behavioral pattern where medical students doubt their abilities and are constantly afraid of being exposed as a fraud. Imposter syndrome might occur due to unrealistic expectations in this highly competitive medical profession.

## INTRODUCTION

Medical Student Syndrome (MSS) is a type of hypochondriacal concern causing health-related anxiety and encompasses the fear in medical students regarding the diseases they are studying.^1,2^ The inexperienced medical students are prone to develop a fear of medical conditions they learn about which is known as "Medical Student Syndrome".^2^

Imposter syndrome is the behavior pattern where people doubt their ability and are constantly afraid of being exposed as a fraud.^3^ It is the consequence of unrealistic and unsustainable expectations of competency.^4^ In the process of absorbing vast knowledge and developing clinical skills, medical students are highly prone to develop this syndrome.

## ETIOLOGY

"Wait...I have heavy periods and it's also painful. Do I have a uterine fibroid or is it Endometriosis?", "I am thirsty too often and pee a lot, do I possibly have Diabetes mellitus?". These are some examples of when medical students seem to self-diagnose themselves which may lead to unnecessary psychological worrying or even hypochondriasis. The cause of symptoms may be something minor or absolutely nothing at all but medical students are convinced of their self-diagnosis which can lead to frequent hospital visits. Regular exposure to new information about diseases makes them wonder whether they also have the disease. Exposure to incomplete information as they say "little knowledge is a dangerous thing" can convince medical students to believe they have developed the disease.

Medicine is a super competitive field where the depth of knowledge is ever-expanding and doctors and medical students are under constant pressure to keep up. Medical students relentlessly focus on the gaps in their knowledge and feel that they are just "pretending" to fit in.^5^ Rigorous academic standards and perfectionistic culture in the medical field are responsible for the high prevalence of imposter syndrome in medical students.^6^ Students in general and particularly medical students tend to compare themselves to their peers mainly in terms of academics which takes a toll on their confidence and leads to imposter syndrome.

## THE UBIQUITY OF THESE SYNDROMES

This unique type of hypochondriacal concern is quite common and was found in about 70% of medical students.^7^ According to a study in Saudi Arabia, nonmedical students had higher visits to doctors compared to medical students but fear of the disease was more common in medical students.^1^ But bigger and better research is required for good evidence regarding the prevalence of Medical Student Syndrome.

Studies show that imposter syndrome is prevalent in about 22-46% of medical students with a higher prevalence in females.^8^ But the actual prevalence is quite high and is yet to be found.

## SPECTRUM AND CONSEQUENCES

Everybody seems to be concerned about their health once in a while, especially in this internet era where even lay people self-diagnose themselves through Google first before going to the healthcare professional which is also known as "Google syndrome", or "cyberchondria".^9^ But when it comes to medical students, they come across common as well as some rare diseases either in textbooks, lectures, or clinics regularly which makes them vulnerable to health-related anxiety even when they are completely fine.

It can range from simple psychological fear to hypochondriacal tendencies with frequent hospital visits. But it shouldn't be supposed that all medical students who come for consultation are going through 'Medical Student Syndrome'. There have been occasions where medical students have been dismissed as having Medical Student Syndrome who turned out to have serious illnesses. Medical Student Syndrome is also known as the third-year syndrome as it is most prevalent in students just beginning their clinical postings.

Imposter syndrome on the other hand can lead to stress, low self-esteem as well as anxiety, and other mental health issues in medical students. Some high-performing medical students may try to work harder than their bodies can handle leading to burnout. Low self-belief and confidence may have a negative impact on one's life which may take a long time to reconstruct.

## OVERCOMING MEDICAL STUDENT SYNDROME AND IMPOSTER SYNDROME

Studying illnesses in-depth and expanding clinical knowledge can somewhat help to prevent this syndrome. Accepting this syndrome as a fairly common condition and embracing it is another way to slowly overcome it. One cannot completely overcome this syndrome but has to learn to live with it with minimum fear. On a positive note, getting this syndrome can help medical students become compassionate doctors and sympathetic toward patients who research their symptoms.

The first step to overcoming imposter syndrome is accepting and normalizing it as a part of learning. Recognizing and celebrating progress along the way rather than focusing on minor shortcomings is a way to overcome it. Shifting focus from "all my colleagues are talented except me" to "I am going to benefit so much from my talented colleagues" can help prevent imposter syndrome and burnout. Another way to overcome imposter syndrome is by remembering you are there for a reason and you fully deserve it.
